# 2,4-D transport and herbicide resistance in weeds

**DOI:** 10.1093/jxb/erw199

**Published:** 2016-05-28

**Authors:** Burkhard Schulz, Kabelo Segobye

**Affiliations:** Plant Science and Landscape Architecture, University of Maryland, College Park, MD 20742, USA

**Keywords:** ABCB transporters, auxin mimics, Brassicaceae, herbicide resistance, translocation, weeds.

**As new herbicide-resistant crops come to market, it can be assumed that there will be a dramatic increase in use of 2,4-D and dicamba. Weed resistance could follow suit, and so a better understanding of the mechanism is urgently needed. In this issue of *Journal of Experimental Botany* (pages 3223–3235), Goggin *et al.* report on their analysis of 2,4-D resistance in wild radish. An elegant combination of physiological and biochemical studies led the authors to identify impaired transport of the herbicide as the cause of resistance.**

Auxinic herbicides such as clopyralid, picloram, dicamba and, above all, 2,4-D – one of the first widely used herbicides – have been used as effective weed control agents since the introduction of 2,4-D herbicides in 1945 (Smith, 1989). The importance of weed control using herbicides cannot be over-estimated. The yield loss potential among all crops without weed control is about 37% (Oerke, 2006); through the use of effective herbicides, these losses have been reduced to about 9% through all cropping systems. Nevertheless, this still means that global agriculture is suffering yield losses worth at least $300 billion each year (calculated using FAOSTAT, 2013: www.faostat.fao.org). Weed control without effective herbicide programs would substantially increase the cost of production, as the input of manual labor would need to be increased. In addition to higher production costs, farmers would also see reduced soil health, greenhouse gas emission and increased soil erosion, as effective no-till agriculture would be nearly impossible. Despite its decades-long worldwide use, resistance against 2,4-D has been found in only 28 different weed species, although the first cases had already been reported in wild carrot (*Daucus carota*) and spreading dayflower (*Commelina diffusa*) in 1957 (Switzer, 1957; Hilton, 1957; Heap, 2016).

The well-known workhorses of the herbicide world, 2,4-D and dicamba, recently became the ‘talk of the agricultural world’ once again. Glyphosate, which came to dominate world herbicide markets after the introduction of transgenic glyphosate-resistant crops in the US from 1996 to 1998 (soybean, maize and cotton) became the epicenter of a rampant resistance epidemic due to overreliance and repeated use. This prompted farmers and the agricultural industry to search for alternative weed-control strategies. Two herbicide producers developed 2,4-D- and dicamba-resistant crops using bacterial resistance genes (Dow AgroSciences with Enlist^®^ and Monsanto with Roundup Ready2 Xtend^®^, respectively) (Behrens *et al.*, 2007; Wright *et al.*, 2010). These new herbicide-resistant crops are now market-ready and it can be assumed that their release will lead to a dramatic increase in use of 2,4-D and dicamba. However, concerns have already been voiced that this might also lead to an increase in 2,4-D resistance in weeds (Egan *et al.*, 2011). It can only be hoped that lessons from the rapid spread of glyphosate resistance have been learned and a similar scenario can be avoided with the use of these new herbicide tolerant crops.

## 2,4-D-resistant weeds

The paper by Goggin *et al.* (2016) shows in very convincing fashion how resistance to 2,4-D in previously described resistant biotypes of wild radish (*Raphanus raphanistrum*) could have evolved (Box 1). The herbicidal mechanism of action of 2,4-D is considered to be activation of the auxin receptor system (TIR1 and related receptor proteins), which results in permanent up-regulation of auxin responses in plants. These include changes in the actin cytoskeleton, followed by up-regulation of the plant hormones ABA and ethylene, and high production levels of reactive oxygen species (ROS). In the end, 2,4-D treatment results in cell wall reorganization, membrane leakage and cell death.

**Box 1. 2,4-D-resistance in wild radish**

The characteristic wild radish inflorescence in the glasshouse (in this example there is color variation – flowers are usually yellow or white). 2,4-D-resistance in wild radish was discovered in large-scale herbicide resistance surveys (Walsh *et al.*, 2007; Owen *et al.*, 2015). The present research takes us an important step further on, through characterizing the resistance mechanism. Courtesy of Dr Danica Goggin.

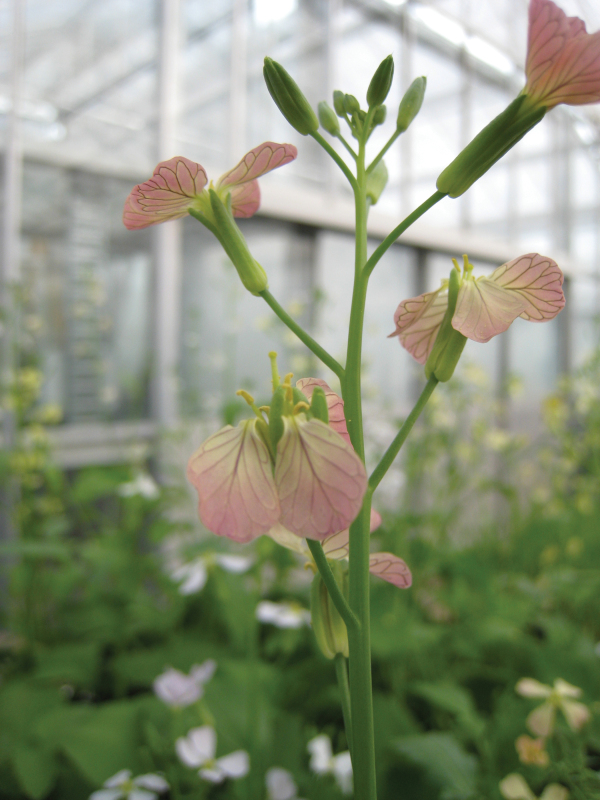

The authors found that ^14^C-radiolabelled 2,4-D is not effectively transported throughout the resistant plant after uptake into the leaf. Phloem loading and transport is clearly impaired in resistant biotypes, leading to localized retention of 2,4-D, mostly in treated leaves blades. The authors went the extra mile to support their hypothesis of a possible auxin transporter mutation being responsible for resistance by clearly ruling out other obvious alternative mechanisms such as 2,4-D metabolism, uptake inhibition and vacuolar sequestration. These have all been found to cause herbicide resistance in other weed species. Additional corroboration came from inhibitor studies using the polar auxin transport inhibitors 1-naphthylphthalamic acid (NPA), 2,3,5-triiodobenzoic acid (TIBA) and ABCB-specific inhibitors such as verapamil and valspodar, which mimic the 2,4-D resistant phenotype. Taking all these data together Goggin *et al.* (2016) make a strong case for their hypothesis of transport inhibition as the cause of 2,4-D resistance.

## Impaired transport

Auxin transporting ATP-binding cassette (ABC) transporters of the B subclass (Martinoia *et al.*, 2002), which have been shown to facilitate polar auxin transport in plants, are the ‘usual suspects’ if auxin transport is involved in herbicide resistance. These plasma membrane transporters are often associated with transport-regulating proteins (TWD1) and auxin membrane facilitators (PINs) to form effective auxin transport complexes in the plasma membrane (Geisler *et al.*, 2003; Bouchard *et al.*, 2006; Zažímalová *et al.*, 2010). It is likely that mutations in members of this auxin transport apparatus can impair the transport of auxinic herbicides such as 2,4-D, as described in Goggin *et al.* (2016). Testing of transport activities with differentially acting inhibitors (NPA acts on ABCB transporters, TIBA acts on PIN facilitators) indicates that the picture of transport inhibition at the molecular level might be more complicated than just a single gene mutation. Indeed, sequencing data from the ABCB gene family and genes of typical ABCB-interacting proteins from resistant and sensitive plants might already indicate how 2,4-D-resistance came about in these wild radish biotypes.

In most cases of resistance to 2,4-D and auxinic herbicides, details of the mechanisms of resistance are not known. Increased absorption of 2,4-D (Kohler *et al.*, 2004), reduced translocation (Weinberg *et al.*, 2006), increased metabolism of 2,4-D (Hagin *et al.*, 1970) and differential binding to auxin-binding proteins (Webb and Hall, 1995) have all been implicated with herbicide resistance. However, reading the published 2,4-D resistance literature with an eye on possible auxin transport impairment shows that similar mechanisms to that described by Goggin *et al.* (2016) might also be the cause of 2,4-D resistance in other cases (Riar *et al.*, 2011; Rey-Caballero *et al.*, 2016).

Elucidating the molecular basis of resistance either at the protein or gene expression level can help the development of novel strategies to overcome a characterized herbicide resistance. ABCB transporters are a rather large subfamily in plants and several members are able to transport auxinic compounds. It is certainly feasible that up-regulation of some members of the family could compensate for the defect in ABCBs responsible for 2,4-D resistance. In Arabidopsis, ABCB4 is the most likely member of the family to transport 2,4-D (Kubes *et al.*, 2012), but the makeup of the wider ABCB family is not yet known in most weed species due to non-existing or insufficient sequence information. Detailed knowledge of expression and activation patterns of these transporters is required. Nevertheless, the findings of Goggin *et al.* (2016) can lead the way in understanding resistance to a very potent herbicide in wild radish, and provide clues as to the way forward in other weed species.

## Family connections

This publication is a nice example of how results achieved using Arabidopsis can inform research in agronomically important plants. Certainly knowledge of auxin biology gathered in the model has informed the process of hypothesis formulation. The fact that wild radish and Arabidopsis both belong to the same family, Brassicaceae, is also likely to ease the analysis of comparative sequence data and could speed up the discovery process in deciphering the molecular basis of 2,4-D resistance in this species.

Agricultural research, especially within the Brassicaceae, has already benefited from basic research performed in Arabidopsis. For example, the molecular basis of the cauliflower phenotype in the cultivated garden variety *Brassica oleracea* has been determined in Arabidopsis (Kempin *et al.*, 1995); and *Camelina sativa* was developed as a potential biofuel and industrial oil crop with the help of Arabidopsis research (Nguyen *et al.*, 2013). Many other examples of trait identification in unrelated crop species based on data from Arabidopsis have also been reported, even involving members of the ABCB gene family (Multani *et al.*, 2003).

Many scientists are convinced that we will see the swan song of the ‘easy herbicide age’ in agriculture as new herbicides with new modes of action are unlikely to be released in the coming years. This makes ever-more spreading herbicide resistance a pressing problem for crop production and will pose great challenges for food security as a growing global population will have greater demand for food supplies. No other pest has a more detrimental effect on crop yields than weeds (Oerke, 2006). The molecular understanding of herbicide resistance mechanisms is still in its infancy. However, recent progress promises a better understanding and provides more options for developing informed strategies to deal with the problem in weeds, such as use of RNAi technology to overcome resistance (Hoemann, 2015).
